# Polymyxin Resistance in Clinical Isolates of *K. pneumoniae* in Brazil: Update on Molecular Mechanisms, Clonal Dissemination and Relationship With KPC-Producing Strains

**DOI:** 10.3389/fcimb.2022.898125

**Published:** 2022-07-15

**Authors:** Orlando C. Conceição-Neto, Bianca Santos da Costa, Leilane da Silva Pontes, Melise Chaves Silveira, Lívia Helena Justo-da-Silva, Ivson Cassiano de Oliveira Santos, Camila Bastos Tavares Teixeira, Thamirys Rachel Tavares e Oliveira, Fernanda Stephens Hermes, Teca Calcagno Galvão, L. Caetano M. Antunes, Cláudio Marcos Rocha-de-Souza, Ana P. D. Carvalho-Assef

**Affiliations:** ^1^ Laboratório de Pesquisa em Infecção Hospitalar (LAPIH), Instituto Oswaldo Cruz - Fundação Oswaldo Cruz (FIOCRUZ), Rio de Janeiro, Brazil; ^2^ Faculdade de Medicina, Universidade Estácio de Sá (UNESA), Rio de Janeiro, Brazil; ^3^ Laboratório de Genômica Funcional e Bioinformática (LAGFB), Instituto Oswaldo Cruz-FIOCRUZ, Rio de Janeiro, Brazil

**Keywords:** polymyxin resistance, *Klebsiella pneumoniae*, antibiotic resistance, mcr gene, PhoPQ and PmrAB

## Abstract

In Brazil, the production of KPC-type carbapenemases in Enterobacteriales is endemic, leading to widespread use of polymyxins. In the present study, 502 *Klebsiella pneumoniae* isolates were evaluated for resistance to polymyxins, their genetic determinants and clonality, in addition to the presence of carbapenem resistance genes and evaluation of antimicrobial resistance. Resistance to colistin (polymyxin E) was evaluated through initial selection on EMB agar containing 4% colistin sulfate, followed by Minimal Inhibitory Concentration (MIC) determination by broth microdilution. The susceptibility to 17 antimicrobials was assessed by disk diffusion. The presence of *bla*_KPC_, *bla*_NDM_ and *bla*_OXA-48-like_ carbapenemases was investigated by phenotypic methods and conventional PCR. Molecular typing was performed by PFGE and MLST. Allelic variants of the *mcr* gene were screened by PCR and chromosomal mutations in the *pmrA*, *pmrB*, *phoP*, *phoQ* and *mgrB* genes were investigated by sequencing. Our work showed a colistin resistance frequency of 29.5% (n = 148/502) in *K. pneumoniae* isolates. Colistin MICs from 4 to >128 µg/mL were identified (MIC_50_ = 64 µg/mL; MIC_90_ >128 µg/mL). All isolates were considered MDR, with the lowest resistance rates observed for amikacin (34.4%), and 19.6% of the isolates were resistant to all tested antimicrobials. The *bla*_KPC_ gene was identified in 77% of the isolates, in consonance with the high rate of resistance to polymyxins related to its use as a therapeutic alternative. Through *XbaI*-PFGE, 51 pulsotypes were identified. MLST showed 21 STs, with ST437, ST258 and ST11 (CC11) being the most prevalent, and two new STs were determined: ST4868 and ST4869. The *mcr-1* gene was identified in 3 *K. pneumoniae* isolates. Missense mutations in chromosomal genes were identified, as well as insertion sequences in *mgrB*. Furthermore, the identification of chromosomal mutations in *K. pneumoniae* isolates belonging from CC11 ensures its success as a high-risk epidemic clone in Brazil and worldwide.

## Introduction

Enterobacteriales adaptability is as impressive as their pathogenicity in humans and animals. While they are ubiquitous in nature and are part of the host´s normal microbiota, they are related to the development of serious diseases. Enterobacteriales are isolated in about 50% of patients with Healthcare-Acquired Infections (HAIs) and comprise 80% of all isolates among Gram-negative bacteria (Jorgensen et al., 2015). In 2017, the World Health Organization ranked Enterobacteriales bacteria as a critical priority for research and development of new antimicrobials, especially for carbapenemase-producing strains (WHO, 2017). In the context of HAIs, the species Klebsiella pneumoniae is noteworthy due to its prevalence and ability to acquire resistance (Trecarichi and Tumbarello, 2017). When considering carbapenemase-producing strains, mortality rates can exceed 35%, mainly in bloodstream infections (Tumbarello et al., 2012; Capone et al., 2013; Giacobbe et al., 2015). The epidemiology accessed through MLST techniques allowed the identification of Clonal Complex 11 (CC11, also referred to as CC258), which includes ST258 and other related STs (such as ST11 and ST340), epidemic *K. pneumoniae* clones that carry a wide variety of genetic determinants, related to antimicrobial resistance. Global spread of the *K. pneumoniae* carbapenemase (KPC) enzyme, for example, has been credited to ST258 (Samuelsen et al., 2009).

Infections caused by carbapenem-resistant *K. pneumoniae* (CRKP) are associated with high mortality rates and are often treated with polymyxins (Falagas et al., 2014). The target of polymyxins is the outer membrane of Gram-negative bacteria, due to an electrostatic attraction existing between the negative charge of phosphate groups present in lipid A of bacterial LPS, and the positive charge of L-alpha-gamma-diaminobutyric acid (L-α-γ-Dab) residues existing in the polymyxin molecule. Although LPS is the initial target of these drugs, the exact mechanism of action leading to bacterial cell death is not fully elucidated (Poirel et al., 2017). It has been proposed that polymyxins could inhibit metabolic pathways associated with cellular respiration (Hill et al., 2014), or even modify bacterial plasma membrane morphology and cell aggregation pattern (O’Driscoll et al., 2018).

Empirical prescription and the use of polymyxins as a therapeutic option for CRKP have been associated with increased rates of polymyxin resistance. Resistance has been described in several genera of Enterobacteriales, such as *Klebsiella, Escherichia, Enterobacter* and *Salmonella*. It is mainly based on the modification of the charge of LPS, by substitution or addition of cationic molecules, especially 4-amino-4-deoxy-L-arabinose (L-Ara4N) and phosphoethanolamine (pEtN) (Sampaio and Gales, 2016; Bartolleti et al., 2016). LPS modifications is a result of constitutive activation of genes encoding enzymes that add L-Ara4N and pEtN to lipid A. In turn, constitutive expression of these enzymes is usually a result of mgrB inactivation (by mutation or by IS element insertion in its coding sequence or promoter region) (Cannatelli et al., 2014; Jayol et al., 2015; Cheng et al., 2015; Jayol et al., 2015), as the MgrB product represses PhoQ activity. Second to mgrB inactivation in causing resistance are missense mutations leading to amino acid substitutions in two component systems (TCSs) PhoPQ and PmrAB, which change their activities, resulting in LPS modification (Poirel et al., 2017).

Plasmids or other mobile genetic elements carrying genes encoding LPS-modifying enzymes have been increasingly identified. These mcr genes exist in numerous allelic variants, and although still rare, they have been described worldwide (Liu et al., 2016; Wang et al., 2020), including in strains susceptible to polymyxins (Wang et al., 2020).

Therefore, to understand the genetic determinants of resistance to polymyxins, it is necessary to identify and characterize them.

## Material and Methods

### Clinical Strains and Screening for Polymyxin-Resistant Isolates

We evaluated 502 non-duplicate Klebsiella pneumoniae clinical isolates (blood, urine, tracheal aspirate, rectal swab, catheter tip, sputum, tissue fragment and wound) received by Laboratório de Pesquisa em Infecção Hospitalar (LAPIH - FIOCRUZ) from January to September 2016 from four Brazilian regions in eight states: Northeast (Maranhão [MA], Piauí [PI], Sergipe [SE]); Central-west: Goiás [GO]; Southeast (Rio de Janeiro [RJ], Minas Gerais [MG], Espírito Santo [ES]) and South (Rio Grande do Sul [RS]). LAPIH is located at Oswaldo Cruz Institute (IOC-FIOCRUZ) and is part of the national surveillance network for the prevalence of bacterial resistance, coordinated by the National Health Surveillance Agency (ANVISA). Hospitals and LACENs (public health central laboratories in different states) voluntarily sent the isolates to LAPIH for confirmation of identification at the species level and determination of the molecular mechanisms associated with resistance to antimicrobials. The isolates came from hospitalized patients and were known to be resistant to carbapenems (or with suspected phenotype).

Species identification was confirmed using conventional biochemical tests (Koneman et al., 2010; Jorgensen et al., 2015). A polymyxin resistance screening was performed, where isolates were inoculated simultaneously on Eosin Methylene Blue agar (EMB) (Oxoid, UK) and on Poly-EMB agar [EMB (Oxoid, UK) + 4 μg/mL colistin sulfate (Sigma-Aldrich, USA)]. For this, a bacterial suspension with an optical density of 0.5 McFarland standard (1.5 × 10^8^ CFU/mL) was prepared and 1 μL (~1.5 × 10^5^ CFU) was plated on the culture media.

Confirmation of colistin resistance was assessed in duplicate using homemade broth microdilution method and interpreted according to CLSI (2020). E. coli ATCC 25922, susceptible to colistin, was used as Quality Control (QC). *Proteus mirabilis* CCBH22517 (intrinsically resistant to polymyxins) and E. coli CCBH23595 (mcr-1-positive, MICcolistin= 4 μg/mL) were also utilized as reference strains.

### Determination of the Antimicrobial Susceptibility Profile

Susceptibility of colistin-resistant *K. pneumoniae* isolates to 17 antimicrobials was performed and interpreted using the disk-diffusion method, according to the CLSI guideline (CLSI, 2015): Amikacin (30 μg), Gentamicin (10 μg), Tobramycin (10 μg), Amoxicillin/clavulanate (20/10 μg), Piperacillin/tazobactam 100/10 μg), Cefoxitin (30 μg), Ceftriaxone (30 μg), Ceftazidime (30 μg), Cefepime (30 μg), Aztreonam (30 μg), Ertapenem (10 μg), Imipenem (10 μg), Meropenem (10 μg), Tetracycline (30 μg), Ciprofloxacin (5 μg), Chloramphenicol (30 μg) and Trimethoprim/sulfamethoxazole (1.25/23.75 μg).

The MIC for ceftazidime/avibactam (CAZ/AVI), a 3rd generation cephalosporin combined with an inhibitor of serine reactive β-lactamases (classes A, C and D) (Krishnan et al., 2015), was determined by E-test (Biomérieux, France). Because all isolates showed MDR profile, we selected the chronologically oldest of each pulsotype determined by PFGE (cited below), totaling 51 isolates. To interpret the results, the breakpoints established by CLSI (2020) were used.

*E. coli* ATCC 25922 and *P. aeruginosa* ATCC 27853 were used as quality controls in both methods.

### Determination of Genetic Diversity in Polymyxin Resistant Isolates

Molecular typing by pulsed-field gel electrophoresis (PGFE) was performed for colistin-resistant *K. pneumoniae* isolates according to the protocol described by Ribot et al. (2006), with adaptations (Ribot et al., 2006). The dendrograms were made using the Bionumerics v.6.6 (Applied-Maths), with 1.0% optimization and 1.5% tolerance. The minimum Dice index of 80% was defined for the inclusion of isolates in the same clonal group.

MLST was performed on at least one representative sample of each clonal group identified by PFGE (51 isolates), using the same chronological selection criteria described for CAZ/AVI testing. For this, we used Protocol No. 2 of the MLST scheme for *K. pneumoniae*, whose database is hosted at the Pasteur Institute (France) and available on the institution’s website (http://www.pasteur.fr/mlst/). The amplified DNA fragments were purified using the GFX PCR DNA Purification Kit (GE Healthcare, USA), following the manufacturer’s instructions. PCR products were sequenced using the BigDye Terminator v3.1 Cycle Sequencing Kit (Applied Biosystems) and the sequences were analyzed by the Sequencing Analysis 5.3 software (Applied Biosystems).

### Identification of Carbapenemase-Coding Genes

The detection of the blaKPC, blaNDM and blaOXA-48-like genes was performed using Polymerase Chain Reaction (PCR) in all isolates, using the primers and amplification conditions previously described (Yigit et al., 2001; Poirel et al., 2010; Nordmann et al., 2011). As quality controls, the *K. pneumoniae* CCBH6556 (blaKPC-positive), K. quasipneumoniae CCBH16302 (blaNDM-positive) and *K. pneumoniae* CCBH10079 (blaOXA-48-like-positive), all obtained from the CCBH collection (http://ccbh.fiocruz.br/) were used.

### Investigation of the Main Genes Related to Polymyxin Resistance

To search for plasmid genes *mcr-1, mcr-2, mcr-3, mcr-4* and *mcr-5*, a multiplex PCR was performed, using the primers and conditions described by Rebelo et al. (2018) (Rebelo et al., 2018). As controls, *E. coli* CCBH20180 (*mcr-1*-positive), E. coli CCBH35182 (*mcr-2*-positive), *E. coli* CCBH35183 (*mcr-3*-positive), *E. coli* CCBH35184 (mcr-4-positive) and *E. coli* CCBH25606 (*mcr-5*-positive) were used.

Genes *pmrA, pmrB, phoP, phoQ* and *mgrB*, described as associated with resistance to polymyxins (Cheng et al., 2010; Olaitan et al., 2014) were amplified using specific oligonucleotide primers in a conventional PCR reaction (Jayol et al., 2014). The amplified DNA fragments were purified using the GFX PCR DNA Purification Kit (GE Healthcare, USA), following the manufacturer’s instructions. Sequencing was performed in a 3730xl DNA Analyzer capillary sequencer (Applied Biosystems) using the BigDye Terminator v3.1 Cycle Sequencing Kit (Applied Biosystems) and the sequences were analyzed with Sequencing Analysis 5.3 software (Applied Biosystems). The sequences obtained for each of the genes were analyzed for integrity and presence of mutations in the Geneious v.1.6.8 software (Biomatters Ltda), through reference mapping with sequences extracted from the complete genome of the *K. pneumoniae* subsp. pneumoniae MGH 78578 (GenBank: NC_009648.1). The ISFinder (https://isfinder.biotoul.fr/) and BLAST (https://blast.ncbi.nlm.nih.gov/) tools were used to identify insertion sequences and other mobile genetic elements in the analyzed genes.

### Mapping Mutations in TCS Proteins

ClustalOmega (Madeira et al., 2019) was used to align the protein sequences in Protein Data Bank (PDB) structures 6RFV (histidine kinase HK853 in complex with response regulator RR468 receiver domain and adenosine diphosphate (ADP), at pH 7) (Mideros-Mora et al., 2020) and 4S05 (active PmrA in complex with DNA) (Ruppé et al., 2015) with PmrAB and PhoPQ sequences encoded in the genome of the *K. pneumoniae* subsp. pneumoniae MGH 78578 (GenBank: NC_009648.1) (**Supplementary Figures 1**–**3**). The alignments were used to identify the positions in the crystal structures that are equivalent to those found mutated in isolates carrying wild type mgrB. These positions were visualized in the crystal structures using Pymol Schroedinger 11c v2.1.0.

## Results

### Screening of Colistin Resistance and Phenotypic Characterization of Isolates

Initially, 209/502 (41.6%) of the isolates were grown on EMB + 4 μg/mL colistin agar. Upon confirmation by broth microdilution, 148 (29.5%) showed MIC > 2 μg/mL (resistant). MICs for colistin ranged from 4 to >128 µg/mL, with MIC50 = 64 µg/mL and MIC90 >128 µg/mL.

**Figure 1:**
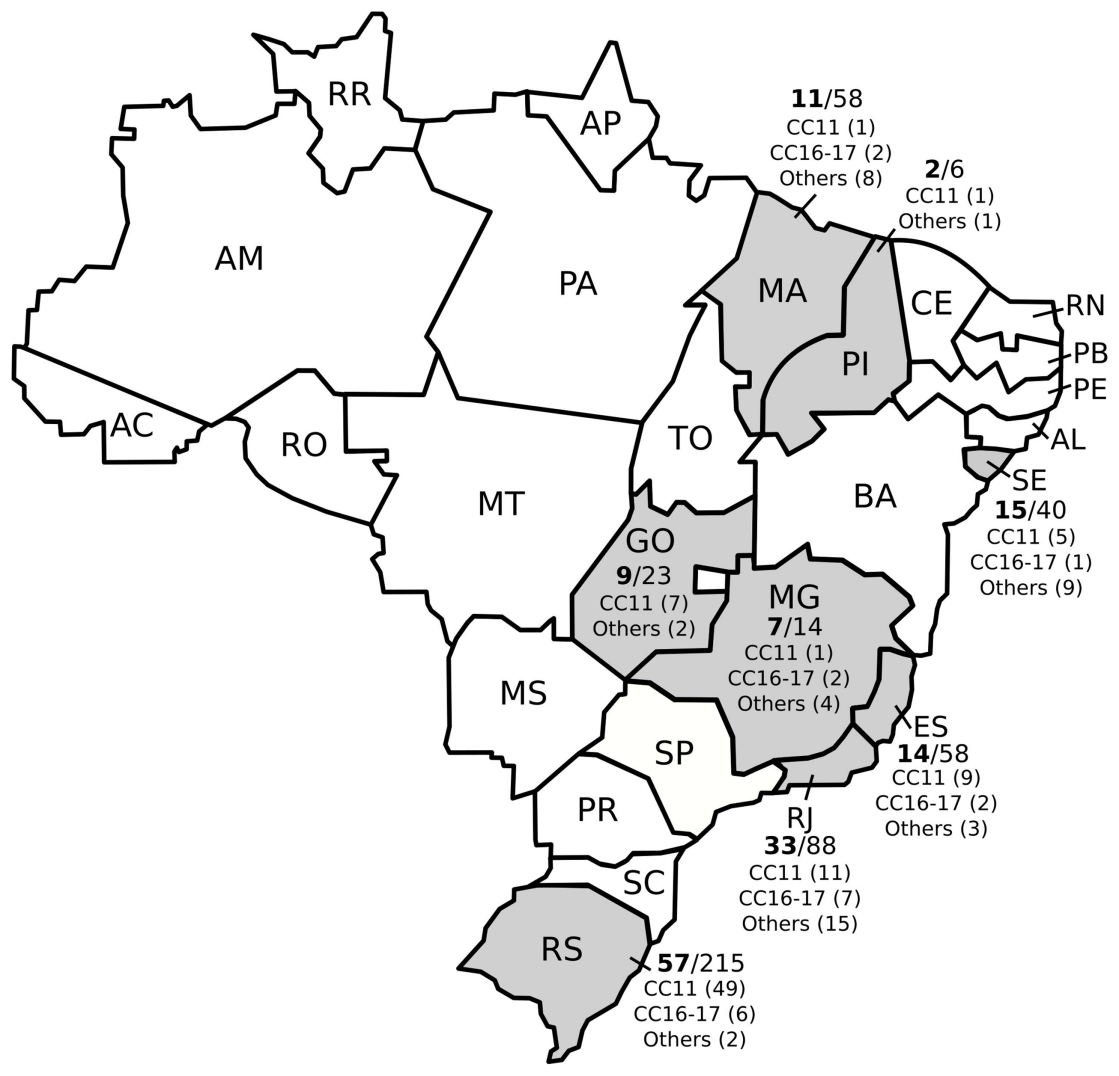
Distribution of colistin resistance and main clonal complexes (CC) among multidrug-resistant Klebsiella pneumoniae isolates from different Brazilian states.

The 502 strains were sent to LAPIH spontaneously by state laboratories, and the frequency of colistin resistance was high in every state. Rio Grande do Sul had the greatest number of colistin-resistant isolates (n=58/215; 27%), followed by Rio de Janeiro (n=32/88; 36.4%), Sergipe (n=15/40; 37.5%), Espírito Santo (n=14/58; 24.1%), Maranhão (n=11/58; 19%), Goiás (n=9/23; 39.1%), Minas Gerais (n=7/14; 50%) and Piauí (n=2/6; 33.3%) (**Figure 1**).

A high rate of resistance was observed for most of the 17 antimicrobials tested by the disk-diffusion method. The lowest resistance rates were observed for the aminoglycosides amikacin (34.4%) and gentamicin (68.2%), as well as for chloramphenicol (66.2%) and tetracycline (64.9%) (**Table 1**).

**Table 1:** Antimicrobial resistance rates tested for 148 colistin-resistant *K. pneumoniae* isolates.

Class	Antimicrobial	Not susceptible ^a^	%
Penicillin/β-lactamase inhibitor	Piperacillin/tazobactam	144	97,3
	Amoxicillin/clavulanate	142	95,9
2nd generation cephalosporin	Cefoxitin	143	96,6
3rd generation cephalosporin	Ceftriaxone	147	99,3
	Ceftazidime	140	94,6
4th generation cephalosporin	Cefepime	144	97,3
Carbapenems	Ertapenem	141	95,3
	Meropenem	130	87,8
	Imipenem	129	87,2
Monobactam	Aztreonam	144	97,3
Aminoglycosides	Tobramycin	119	80,4
	Gentamicin	101	68,2
	Amikacin	51	34,4
Tetracyclines	Tetracycline	96	64,9
Amphenicols	Chloramphenicol	98	66,2
Quinolones	Ciprofloxacin	141	95,3
Folate pathway inhibitors	Sulfamethoxazole/trimethoprim	129	87,2

a A bacterial isolate was considered non-susceptible when it tested resistant or intermediate to the antimicrobial agent, according to the criteria established by Magiorakos et al. (2012).

According to the concept of multidrug resistance defined by Magiorakos et al. (2012) (Magiorakos et al., 2012), all selected *K. pneumoniae* isolates were considered MDR (n=148, 100%), since they were not susceptible to at least one representative of three or more classes of antimicrobials, and 29 (19.6%) were not susceptible to any of the antimicrobials tested by the disk-diffusion method.

The testing of Ceftazidime/Avibactam (CAZ/AVI) in 51 representative isolates selected by the PFGE confirmed that 92.1% (n= 47) were susceptible. MICs ranged from 0.25 to >256 µg/mL (MIC50 = 0.75 µg/mL and MIC90 = 2 µg/mL). All four CAZ/AVI resistant isolates had MIC >256/4 μg/mL and one of them was positive for *bla*_NDM_ gene and two were positive for *bla*_KPC_.

### Genotypic Typing by PFGE and MLST

Analysis of XbaI-PFGE restriction fragments showed 51 distinct clonal groups (“Kp1”-”Kp51”). Kp11 was the most prevalent (n=23, 15.5%), followed by Kp3 (n=12, 8.1%) and Kp14 (n=8, 5.4%). All isolates of Kp11 and Kp3 pulse types were from Rio Grande do Sul. The most heterogeneous distribution was identified in isolates from the state of Rio de Janeiro (17 groups in 33 isolates). We also detected that 4 clonal groups were identified in three or more states: Kp4 (GO, MG, PI, RJ); Kp1 (GO, RJ, SE); Kp2 (GO, RJ, RS) and Kp14 (ES, RJ, RS).

MLST analysis identified 21 different sequence types (STs) in the 51 selected isolates. CCBH22382 (Kp20) and CCBH22381 (Kp36) did not have their ST determined due to loss of viability, as well as CCBH21941 (Kp22), CCBH23167 (Kp26), CCBH22723 (Kp37) and CCBH22404 (Kp41), due to problems in the amplification of the genomic material and the quality of the sequences. These strains were not replaced as the clonal group contained only one representative.

The most prevalent clonal groups determined by PFGE, Kp11, Kp3 and Kp14, were identified by MLST as belonging to ST437, ST11 and ST16, respectively. CCBH21875 (Kp23) and CCBH22680 (Kp35) had new STs assigned by the Pasteur Institute: ST4868 and ST4869, respectively. CCBH21875 (ST4868) is from the state of Maranhão, showed MIC for colistin >128 μg/mL and was negative for the carbapenemase genes studied. The isolate CCBH22680 (ST4869) was from the state of Rio de Janeiro, showed MIC for colistin equal to 64 μg/mL and was positive for the blaKPC gene. Both were recovered from rectal swabs from hospitalized patients and were only susceptible to aminoglycosides and chloramphenicol.

Colistin-resistant CC11 *K. pneumoniae* isolates wereidentified in all states evaluated and 19 pulse types identified in PFGE were associated with CC11 (ST11, ST258 or ST347). ST437 was the most prevalent (56.8%) (**Figure 1** and **Supplementary Table 1**).

### Identification of Frequent Carbapenemases and Colistin Resistance Genes

The *bla*_KPC_ gene was identified in 122 (82.4%) of the isolates, in all Brazilian states studied. The *bla*_NDM_ gene was identified in 3 (2%) isolates from Sergipe classified in different clonal groups, and *bla*_OXA-48-like_ in 2 (1.3%) Kp14 isolates, from Rio de Janeiro and Espírito Santo. No co-production was detected.

Three isolates CCBH22220, CCBH22399 and CCBH22609 were positive for the *mcr-1* gene (2.0%). The other *mcr* genes investigated were not detected. CBH22220 was also positive for the blaKPC gene.

### Chromosomal Mutations Associated with Colistin Resistance

Numerous silent nucleotide changes were identified in *mgrB, pmrA, pmrB, phoP* and *phoQ* genes of the 148 colistin-resistant *K. pneumoniae* isolates. Also, many missense and nonsense mutations were detected, in addition to IS sequences in mgrB and a few insertion-deletion mutations (INDELs) (**Table 2** and **Supplementary Table 1**). The presence of IS102 was detected in the coding region of mgrB in five isolates and, for this element, a probable hotspot was identified, since the insertions occurred at position +90 in CCBH22997, CCBH23000 and CCBH23031, and at position +89 in CCBH22999 and CCBH23001.

**Table 2:** Genotypic profiles constructed according to the set of mutations existing in the studied loci, sequence type defined by the MLST and correlation with the MIC for colistin.

ST	Profile	PmrA	PmrB	PhoP	PhoQ	MgrB	No.	Colistin MIC (g/mL)
ST11	1	L63H	R256G				1	>128
	2		R256G		V27H, P103W, C395A	ISKpn13	1	64
	3		R256G			ISKpn13	1	32
	4		R256G		E397G		1	64
	5		R256G	100_101 ins11pb	V27H, P103W		1	128
	6		R256G			K3T	1	4
	7		R256G			I10T	1	>128
	8					ISKpn13	1	128
ST258	9		R256G			G37S	1	>128
	10		R256G		I88N		1	32
ST437	11		R256G			IS1R	1	32
	12		R256G			S36R	2	16 and 64
	13		R256G		V27H, P103W, C395A	ISKpn26	1	32
	14		R256G		V27H, P103W, C395A	S36R	1	32
	15		R256G		P103W, C395A	S36R	1	32
	16		R256G			IS903B	1	128
	17		R256G		P103W		1	32
	18		R256G		V27H, P103W, C395A		1	32
	19		R256G		L257W Q287K, 878_879insAG		1	>128
	20				765_766insC		1	32
	21					IS903B	1	32
ST76	23		R256G		1077_7078insAG	V1E, K3Stop	1	32
	24		R256G	G121A			1	64
ST15	25				V27H, Y264T		1	64
	26				T276C		1	64
	27					ISKpn26	1	32
ST16	28				V27H, P103W, C395A		1	32
	29				Y265C		1	64
	30				R16A, V27H		1	64
	31				V27H, Y265C		1	64
ST17	32					L8Stop	1	64
ST111	33					K3Stop	1	32
ST147	34		R256G		V27H, F398K	IS903B	1	32
	35		R256G		V27H, F398K	IS102	1	16
	36		R256G			IS102	1	32
ST252	37				V27H, P103W, C395A		1	8
ST987	38		R256G		1077_1078insAG		1	4
ST477	39				A325V		1	8
ST48, ST147 ST340, ST437	40		R256G			IS903B	7	32 to >128 ^a^
ST11, ST437	41		R256G		V27H		3	16 to 128 ^b^
ST3228, ST437	42		R256G		V27H, P103W		2	16
ST48, ST258	43		R256G			ISKpn26	2	64 to >128
Others ^c^	44		R256G				46	4 to >128

only isolates in which missense mutations were identified are represented in this table.

**^a^** Two isolates of ST48 (MIC= 32 μg/mL), two of ST147 (MIC >128 μg/mL), one isolate of ST340 (MIC= 128 μg/mL) and one of ST437 (MIC= 64 μg /mL).

**^b^** Two ST11 isolates (MICs= 16 and 128 µg/mL) and two ST437 isolates (MICs= 64 and 128 µg/mL).

**^c^** This profile includes the STs: ST11, ST15, ST48, ST76, ST258, ST340, ST437, ST477 and ST617.

In 9/148 (6%) of the isolates, the sequences of the five genes studied were identical to that of the reference strain. Additionally, over 57 isolates (38.5%) display only the T246A and/or R256G changes, which are not associated with resistance as they are also found in susceptible isolates (unpublished results). In these isolates, resistance is likely caused by alternative mechanisms, such as insertion sequences in the mgrB promoter or missense mutations in crrAB and crrC (Rodríguez-Santiago et al., 2021).

Currently, it is not known how substitutions in PhoPQ and PmrAB alter TCS function such that transcription of genes encoding LPS modifying enzymes becomes constitutive and leads to resistance. While the details of TCS mechanisms vary, conferring exquisite specificity to kinase/regulator pairs, there are underlying similarities that are shared among homologs (Buschiazzo and Trajtenberg, 2019). Thus, we took an *in silico* approach to visualize the equivalent positions of PmrAB and PhoPQ substitutions in the structural context of a homolog pair. As it is not known how these substitutions affect polymyxin resistance in isolates that also carry inactive mgrB, the analysis only included thirteen missense mutations in phoPQ and pmrAB specific to isolates whose mgrB gene was intact (**Supplementary Table 1**).

**Figure 2:**
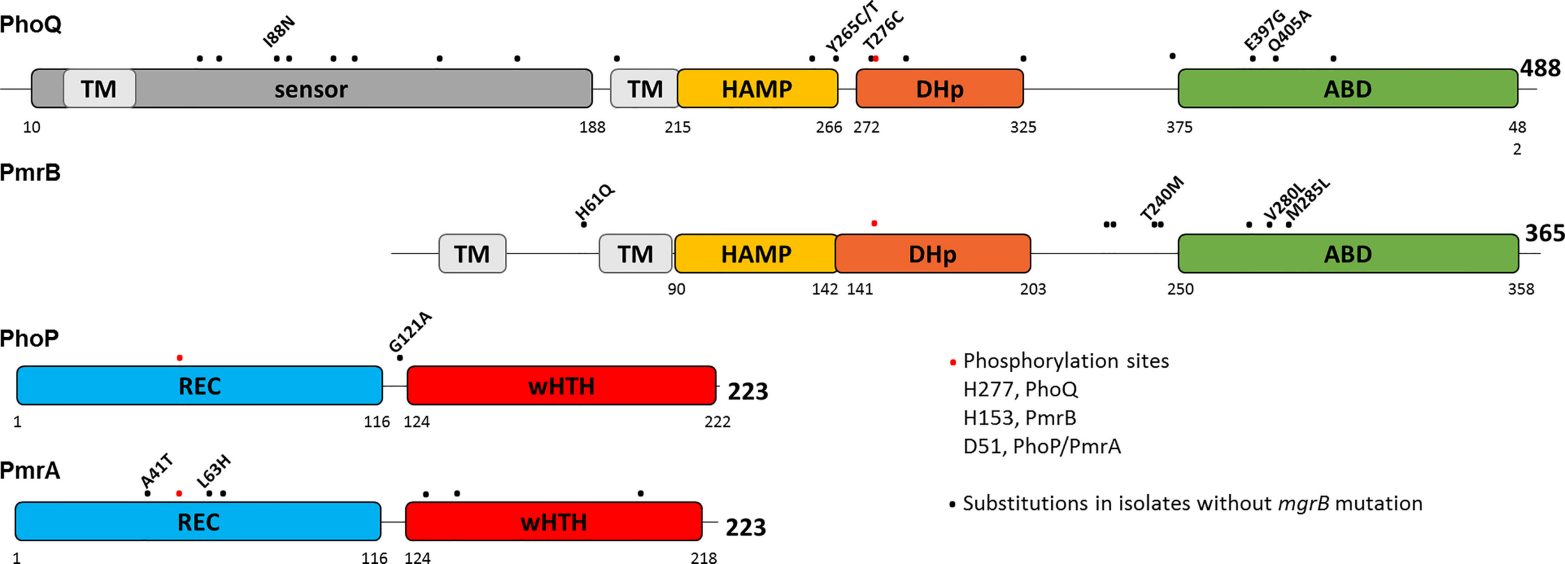
Substitutions in PhoPQ and PmrAB are unevenly scattered across the proteins. Schematic diagram representing the domain structure of PhoPQ and PmrAB according to InterPro. Amino acid positions are indicated for each domain and for the full length protein. Phosphorylated amino acids are shown as red dots: H277 and H153 in PhoQ and PmrB, respectively, and D51 in PhoP and PmrA. Black dots represent amino acid substitutions encoded in isolates carrying intact *mgrB*. Three isolates carry several mutations in PmrAB and PhoQ, which are shown solely as dots and are not explored further (CBB22114: PmrA S64A, N131D, L140Q, E199D; PmrB N105S, A228T, Q232E, I242V, N244S, T246A; PhoQ A69K, Q92K, A106T, E112D, I139V, L163F, V196I, Q424P. CBB22871: PmrB N105S, A228T, Q232E, I242V, N244S, T246A, E272Q; PhoQ L163F, V196I, A325V; CBB23286: PmrB N105S, A228T, Q232E, I242V, N244S, T246A, E272Q; PhoQ R64K, Q92K, A106T, E112D, I139V, L163F, V196I, T372S, Q424P).

The domain organization of PhoPQ and PmrAB as predicted by InterPro (Blum et al., 2021) shows that the substitutions are scattered along the proteins, but a few cluster in a short region of the ATP binding domain (ABD) of PmrB and PhoQ. The dimerization and histidine phosphotransfer (DHp) domain harbors the conserved histidine (H277 and H153 in PhoQ and PmrB, respectively) that is autophosphorylated, and from which phosphotransfer occurs to D51, a conserved aspartate in the receiver (REC) domain of the response regulators (RR) PhoP or PmrA (**Figure 2**).

**Figure 3:**
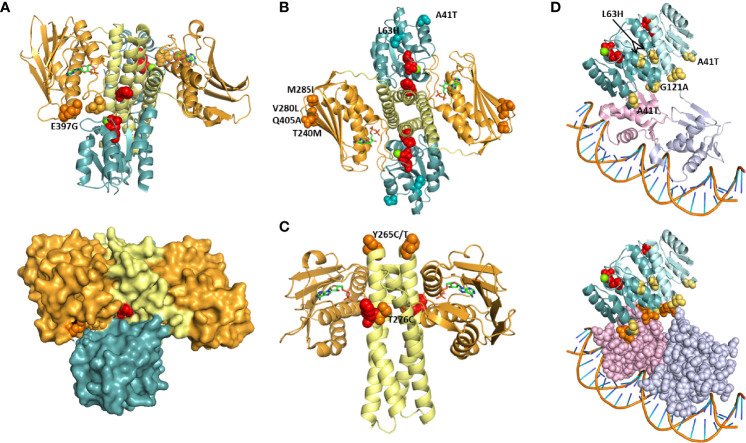
Projection of PmrAB and PhoPQ substitutions on TCS structure. Positions equivalent to those found mutated in colistin resistant isolates were mapped onto structures of a HK/RR complex (A-C; PDB 6RFV) and of PmrA in complex with DNA (D; PDB 4S05). HK853: DHp (yellow) and ABD (orange) domains. RR468: REC (teal). PmrA dimer: REC domains, teal and blue; DBDs, pink and light purple. Red spheres: phosphorylatable residues His260 (HK853), D60 (RR468), D51 (PmrA). ADP is shown as sticks. Green: Mg2+. **(A)** The HK853 position equivalent to E397, K387, is shown as orange spheres. E438 makes a salt bridge with K387 and is shown (light orange spheres). Lower panel: HK-REC interface amino acids P57 and V58 in REC β3-α3 loop are shown as spheres. **(B)** The HK853 and RR468 positions equivalent to PhoQ M285, Q405, PmrB T240, M285, PmrA A41 and L63 are shown as spheres. **(C)** HK853 positions equivalent to PhoQ Y265 and T276 are show as spheres. **(D)** PmrA and PhoP positions found in resistant isolates are shown as yellow spheres. Lower panel: the REC-DBD interface is highlighted by using a space filling representation of amino acids in the DBD. N43 and N120, adjacent to A41 and G121, and amino acids with which they hydrogen bond (Q122, N125 and N176) are shown as orange spheres.

To explore the structural context of PmrB and PhoQ substitutions, we mapped the equivalent residues in the crystal structure of the HK/RR complex HK853/RR468 of *Thermotoga maritima* (**Figure 3**). HK853/RR468 is a model for TCS structure and function studies, with HK853 belonging to the HisKA family and RR468 belonging to the OmpR RR family, as also predicted for PmrAB and PhoPQ. The structures available span only the DHp and ABD domains of HK853 and the REC domain of RR468, such that sensor domain substitutions PmrB H61Q and PhoQ I88N were not included in the analysis. Still, it is worth noting that PmrB H61 is immediately adjacent to a conserved glutamate essential for pH sensing in the *Salmonella* homolog (Perez and Groisman, 2007). Also, I88 is in the vicinity of P83, essential for SafA binding and PhoQ activation in E. coli (Yoshitani et al., 2019).

HK853 K387 is equivalent to PhoQ E397, substituted by a glycine in one of the isolates. Adjacent to these positions is the conserved N box region for ATP binding (N376-Y384 in HK853) (Bem et al., 2015). K387 makes a salt bridge with E438, and this pair of amino acids is at the interface with the REC domain of RR468 (**Figure 3A**). The region contacted in the REC domain is the β3-α3 loop, where the phosphorylated aspartate D51 is located. Additionally, the K387-E438 pair contributes to the closure of the ATP pocket observed in the structure. These observations suggest that the E397G substitution affects phosphotransfer between the ABD and the REC domain by modulating HK-RR interface contacts.

Three positions substituted in PmrB (T240M, V280L, and M285I) and one in PhoQ (Q405A) are equivalent to HK853 amino acids located at the outer edge of the anti-parallel β sheet in the ABD: S355, D399 and G404 (**Figure 3B**). Changes in this surface exposed and peripheral position may change the stability of the sheet, modulating phosphorylation dynamics and enhancing phosphotransfer. It is interesting that PhoQ Q405A and PmrB V280L both align with D399 in HK853 (**Figure S3**), suggesting that substitution at this position is relevant for HK function. The MIC of the isolates bearing these substitutions is 16 μg/mL, except for CCBH23220 (PmrB M285L), with a MIC of >128 μg/mL. PmrB V280L has been described in an isolate with MIC of 64 μg/mL which carried additional mutations (PhoP S86L and PmrB L157P) (Cheng et al., 2015). Position 405 has been previously reported to be substituted by a lysine in a colistin resistant isolate that also carries a substitution at position 406, and whose MIC is 4 μg/mL (Mbelle et al., 2020).

PhoQ T2*7*6C is adjacent to H277 (**Figure 3C**), and its close proximity likely affects phosphorylation kinetics and-or signal transmission through the central α helix made up by the HAMP and DHp domains. PhoQ Y265 was found substituted by cysteine or threonine in three isolates. This residue is equivalent to the first amino acid in the HK853 structure, I247 (**Figure 3C**), and its mutation may impact signal transmission from the periplasmic domain to the ABD. Of the substitutions in this *in silico* analysis, only two are found in mcr-1 carrying isolates: Y265T and T276C, which both have MICs of 64 μg/ml. Two isolates bearing the PhoQ Y265C substitution also have a MIC of 64 μg/mL, such that mcr-1 contribution to resistance is not clear (**Supplementary Table 1**). Increased resistance by expression of a mcr-1 gene in a strain carrying pmrB mutation has been observed in *Escherichia coli* (Jayol et al., 2017), showing that these mechanisms can compound the ability to resist polymyxins at least in some cases.

To enquire about the mechanism of resistance conferred by substitutions in RRs (PmrA, A41T and L63H and PhoP, G121A) these positions were visualized in the PmrA-DNA complex crystal structure (**Figures 3B, D**) (Lou et al., 2015). Phosphorylation of the conserved aspartate in the α3-β3 loop of RRs (in red in **Figures 3B, D**) triggers structural transitions through the REC domain that bring the RRs ability to bind DNA and activate transcription. PmrA L63 is at the end of α helix 3, and this position has been suggested to transmit changes caused by phosphorylation in another RR, Spo0B (Feher et al., 1998), such that its substitution to a histidine may enhance the transition to an active state. RRs bind DNA as dimers, and those of the OmpR family such as PmrA and PhoP bind with each monomer in a different configuration (Gao et al., 2019), with one REC domain mounted onto a DNA binding domain (DBD) while the other “floats” (**Figures 3B, D**). The REC-DBD interface made up of extensive hydrogen bonding, including contribution by N43 and N120 (Lou et al., 2015). These amino acids are adjacent to the substituted positions PmrA A41T and PhoP G121. N43 hydrogen bonds with N176 in the DBD, contributing to the REC-DBD interface. G121 of PhoP is equivalent to N121 in PmrA, which hydrogen bonds with Q122 and N125, contributing to the hinge region that holds the “wing” of the DBD, made up of a three sheeted anti parall β sheet. Thus, it is possible that these two substitutions affect transcriptional activation by modulating REC-DBD interactions that favor the DNA bound configuration.

Four isolates bearing PmrB H61Q, PmrA A41T, or PhoQ Y265C carried additional substitutions (PmrA E57G, PmrB L213M or PhoQ V27H and C395V) (**Table 2**). These additional substitutions were also found in isolates bearing mutations or insertions in mgrB, which suggests that either they represent allelic variations independent of resistance, or that their contribution to resistance is more complex. The MICs of the fourteen isolates carrying the substitutions analyzed by comparison to HK853/RR486 ranged from 4 to >128 μg/mL, with a median of 32 μg/mL. The median for all isolates is 48 μg/mL, indicating a similar MIC distribution.

## Discussion

### Resistance to Polymyxins

Recently, polymyxins were categorized by the WHO as “critically important” antimicrobials, as they fulfill the criteria of being one of the few therapies available to treat serious bacterial infections (WHO, 2017).

Data from the SENTRY Program from 2001 to 2004 showed polymyxin B resistance rates in *K. pneumoniae* of 1.8% (Gales et al., 2006). On the other hand, the increase in resistance to carbapenems in the last decade, caused by the spread of KPC, also increased resistance to polymyxins, as observed in a study carried out in the city of São Paulo: 0% in 2011, 4.8% in 2014 and 27.1% in 2015 (similar to that obtained in the present study, 29.5%) (Bartolleti et al., 2016). In 2013, a study conducted by our laboratory aimed to update the molecular epidemiology of *K. pneumoniae* producing KPC-2 in Brazil identified 15% resistance to polymyxin B in 113 isolates received in 2010 (Pereira et al., 2013). In the current study, we detected a resistance frequency of 29.5% in isolates of the same species received by LAPIH in 2016, reinforcing the need to adopt control measures.

Different rates of resistance to polymyxins in *K. pneumoniae* have been described around the world, mainly associated with resistance to carbapenems: 36.1% (Capone et al., 2013); 28% (Grundmann et al., 2017); 12% (Tumbarello et al., 2012); 9.7% (Sader et al., 2014); 7% (Rossi et al., 2017). Considering that 80.3% of the colistin-resistant *K. pneumoniae* isolates in this study were carbapenemase producers (77% KPC), the main cause of the increase in polymyxin resistance in recent years can be credited to the adoption of colistin as a first-line drug to treat infections caused by carbapenem-resistant Gram-negative bacilli. However, the sample group used in this study came from a voluntary demand by hospitals, therefore it may not represent accurately the rate of polymyxin resistance in Brazil.

### Antimicrobial susceptibility: MDR profile in all isolates, aminoglycosides and CAZ/AVI as remaining therapeutic options

According to our results, the aminoglycoside amikacin and gentamicin are the best therapeutic options for colistin resistant isolates, displaying bactericidal activity against 65.6% and 31.8% of isolates, respectively. Still, 22.3% of the isolates were only susceptible to aminoglycosides, such that they remain an option in antimicrobial therapy against Gram-negative MDR rods not susceptible to carbapenems and polymyxins. A European multicenter study reported 71% susceptibility to aminoglycosides in *K. pneumoniae* isolates obtained between 2010 and 2012 from 23 centers (19 in Italy and 4 in Greece) (Cannatelli et al., 2014). However, lower resistance rates have already been reported in 2013 in Greece (19.7%), which shows the importance of surveillance related to the use of these drugs (Maltezou et al., 2014).

In this multidrug resistance scenario, previous exposure to aminoglycosides has recently been associated with an increased risk of acquiring infections caused by polymyxin-resistant *K. pneumoniae* (da Silva et al., 2020). Our analysis revealed the therapeutic potential of CAZ/AVI, since 92.1% (n= 47/51) of the tested isolates were considered susceptible to the association. Thus, antimicrobial association can be considered an alternative in the treatment of infections whose therapeutic options are extremely limited, as recently found by Kazmierczak and colleagues (2018), who found that 98.2% of the 6719 studied *K. pneumoniae* isolates, collected from 96 medical centers in 18 European countries, were susceptible (Kazmierczak et al., 2018).

### Polymyxin Resistance: Association with Carbapenem Resistance, High MICs and Genetic Determinants

In this study, resistance to high levels of colistin in carbapenem-resistant *K. pneumoniae* isolates were detected, and this pattern was also reported by other authors (Cannatelli et al., 2014; Cheng et al., 2015). This profile of isolates is expected, given the selective pressure to which these strains are exposed in the hospital environment. KPC remains the enzyme most often associated with carbapenem resistance in Brazil, and its national prevalence has been confirmed by other authors (da Silva et al., 2020).

These data emphasize the relationship between the dissemination of genes encoding carbapenemases and the increase in resistance to polymyxins in *K. pneumoniae*, as polymyxins are used as an alternative to non-susceptibility to carbapenems.

We identified three *mcr-1*-positive isolates (2.0%): CCBH22220, CCBH22399 and CCBH22609, belonging to the same pulsotype (Kp21) and ST15, all from southeastern Brazil. CCBH22220 was also a co-producer of KPC. The low frequency of the mcr gene in colistin-resistant *K. pneumoniae* isolates was also observed by other authors (Malli et al., 2018), and a recent bacterial fitness study correlates this fact with a significant fitness cost to maintain the gene carrier plasmid (Nang et al., 2018).

Recently, alanine scanning mutagenesis and FRET were used to identify key residues for MgrB function (Yadavalli et al., 2020). Three of the residues whose change to alanine was found to inactivate MgrB, thus leading to PhoQ constitutive activation, displayed substitutions in isolates in this study (K2R, K3T and G37S; **Supplementary Table 1**). In fact, the functional role of the G37S substitution in polymyxin resistance was described in the original study showing that wild type mgrB expression could restore susceptibility to isolates carrying this substitution (Cannatelli et al., 2014). In previous work conducted by our laboratory in *K. pneumoniae* isolates recovered from rectal swabs from hospitalized patients in 11 Brazilian states (Aires et al., 2016), we identified substitution C28R. Mutation at this same position was also found to abolish MgrB activity (Yadavalli et al., 2020). Importantly, the FRET study mentioned above identified the essential role of amino acid W20 in the interaction with PhoQ, and mutations in this position have been identified in polymyxin resistant invasive K. pneumonia ST258 isolates associated with high mortality in Brazil (Roch et al., 2022).

The overall trend observed in our comparative analysis is that TCS substitutions associated with polymyxin resistance occur nearby functional regions. The sensor domain substituted positions (PmrB H61Q and PhoQ I88N) are nearby amino acids necessary for signal sensing or activation (Perez and Groisman, 2007; Yoshitani et al., 2019). PhoQ DHp domain substitution T276C is immediately adjacent to the phosphorylatable H277, being thus poised to alter the local structure with impact on the transition between phosphotransferase and phosphatase configurations. PhoQ E397G, two positions down from the N box for ATP binding and at the interface with the REC domain, may modulate phosphorylation and RR binding. The clustering of PhoQ Q405 and PmrB T240, V280 and M285 at the edge of the antiparallel beta sheet in the ABD suggests a common mode of altering HK function. Visualization of the structural context of the positions substituted in resistant isolates goes towards understanding the features affected and proposing hypothesis that can be tested to show the biochemical basis of resistance.

The genotypic profiles constructed from the association of missense mutations allowed us to conclude that, in our isolates, there was no correlation between mutations alone or together and the MIC range for colistin.

### Clonal Diversity of Polymyxin-Resistant *K. pneumoniae*: Prevalence of CC11, Including High-Risk Epidemic Clone ST258

In the present work, 4 STs belonging to *K. pneumoniae* clonal complex 11 (CC11) were identified in the 51 isolates genotyped by MLST: ST11 (9.8%), ST258 (3.9%), ST340 (5.9%) and ST437 (17.6%). Considering all the isolates associated with pulse types in which CC11 was determined, 56.8% of the clinical isolates studied were related to this clonal complex. These are high-risk epidemic clones associated with the spread of KPC in Brazil and worldwide (Bowers et al., 2015; Dong et al., 2018). The *bla*_KPC_ gene was identified in 91.7% of these isolates. The three isolates resistant to carbapenems and to the ceftazidime/avibactam association (CCBH22684, CCBH22760 and CCBH23616) belong to ST437, ST11 and ST258, respectively. This multidrug resistance scenario, composed of a high number of CC11 isolates and a high prevalence of KPC, highlights the importance of increasing control and surveillance measures.

ST258 (CC11) is a high-risk epidemic clone, described in 2009 in the United States (Kitchel et al., 2009). In Brazil, the first description of this clone was performed by Andrade et al. (2011), in the city of Ribeirão Preto, São Paulo (Andrade et al., 2011). However, it was not identified in a previous study by our laboratory that evaluated the epidemiology of this carbapenemase in Brazil (Pereira et al., 2013). Our data show that the increase in frequency, compared to previous studies, is associated with the maintenance of these epidemic clones in Brazilian hospitals, especially ST437 (most frequent) and the other STs of CC11.

It should be highlighted that ST16, a non-CC11 group, was the third most frequent ST in this study (6.8%). This clone was able to acquire carbapenemases such as *bla*_KPC_ and *bla*_OXA-48-like_. This clone was first reported in Brazil by 2013 (Pereira et al., 2013) and was recently associated to an outbreak with high mortality rates (Andrey et al., 2020). Therefore, ST16 spread must be monitored.

As the prevalence of the mcr gene was low, resistance to polymyxins in *K. pneumoniae* seems to be related to the occurrence of mutations in chromosomal genes, which appear due to selection, associated with the progressive use of polymyxins as an alternative to the treatment of infections caused by carbapenem-resistant strains. On the other hand, previous studies have already demonstrated the isolation of colistin-resistant bacteria in healthy individuals not previously exposed to antimicrobials (Olaitan et al., 2014), and this is probably due to spontaneous mutations in the genomic DNA that led to this status.

Although the *mcr-1*-producing *K. pneumoniae* isolates identified in the present study belong to ST15, a clone spread throughout the world (Salloum et al., 2017), apparently there is no predominant epidemic clone described in the literature that is associated with the acquisition of plasmids containing the mcr gene.

## Conclusion

The frequency of resistance to polymyxins in *K. pneumoniae* isolates from eight Brazilian states was similar to what has been reported in other countries. These results are alarming, since they represent a significant increase in relation to previous data from Brazil.

19.6% of the isolates studied were resistant to all antimicrobials tested by the disk-diffusion method, and amikacin proved to be the antimicrobial with the highest activity. Ceftazidime/Avibactam was effective against 94.1% of the tested isolates, proving to be a therapeutic option for colistin-resistant and serine-carbapenemase-producing strains.

Resistance to high levels of colistin was detected, and chromosomal mutations in Two Component Systems proteins were the likely reason, emerging as a result of the selective pressure to which these strains are exposed.

Our data allow us to infer that resistance to polymyxins in *K. pneumoniae* seems to behave similarly to what occurs with resistance to carbapenems mediated by KPC, in view of the majority identification of CC11 strains in our isolates.

In Brazil, there is an emergence in the association of resistance to carbapenems and polymyxins, which has also been observed in the world. This is a critical association, as therapeutic options become scarce, and these microorganisms are generally associated with unfavorable outcomes, with a high mortality rate. For K. pneumoniae, these results present a serious challenge for Brazilian public health.

## Data Availability Statement

The original contributions presented in the study are included in the article/**Supplementary Material**. Further inquiries can be directed to the corresponding author.

## Author Contributions

AC-A conceived the research. OC-N, BS, LS, LJ, IC, TT, MC, and CB were responsible for the execution of phenotypic and molecular tests to detect polymyxin resistance in *K. pneumoniae*. OC-N and MC were responsible for PFGE and MLST analysis. OC-N, FS, TG, and LA were responsible for the analysis of mutations in the genes encoding the polymyxin-resistance. OC-N, AC-A, and CR-d-S performed the data analysis. CR-d-S and TG made the figures. OC-N, AC-A, CR-d-S, and TG wrote parts and edited the complete manuscript. All authors contributed to the article and approved the submitted version.

## Funding

This work was funded by Conselho Nacional de Desenvolvimento Científico e Tecnológico (CNPq), Fundação Carlos Chagas de Amparo à Pesquisa (FAPERJ), Coordenação de Aperfeiçoamento de Pessoal de Nível Superior (CAPES), Institute Pasteur Inter Concerted Pasteurian Actions (ACIP), Instituto Oswaldo Cruz (IOC), and Fiocruz.

## Conflict of Interest

The authors declare that the research was conducted in the absence of any commercial or financial relationships that could be construed as a potential conflict of interest.

## Publisher’s Note

All claims expressed in this article are solely those of the authors and do not necessarily represent those of their affiliated organizations, or those of the publisher, the editors and the reviewers. Any product that may be evaluated in this article, or claim that may be made by its manufacturer, is not guaranteed or endorsed by the publisher.
