# Supplementary material for: Polymyxin Resistance in Clinical Isolates of K. pneumoniae in Brazil: Update on Molecular Mechanisms, Clonal Dissemination and Relationship With KPC-Producing Strains
Source: Front Cell Infect Microbiol. 2022 Jul 15;12:898125. doi: 10.3389/fcimb.2022.898125 (PMC9334684; doi:10.3389/fcimb.2022.898125)
Supplement: Supplementary file 1 [file Image_1.pdf]

```
RR468        MSKKVLLVDDSAVLRKIVSFNLKKEGYEVIEAENGQIALEKLSEFTPDLIVLDIMMPVMD   60
PhoP_Kp      --MRVLVVEDNALLRHHLKVQLQELGHQVDAAEDAREADYYLGEHLPDIAIVDLGLPDED   58
                :**:*:*.*:**: :..:*:: *::*  **:.: *   *.*. **: ::*: :*  *

RR468        GFTVLKKLQEKEEWKRIPVIVLTAKGGEEDESLALSLGARKVMRKPFSPSQFIEEVKHLL   120
PhoP_Kp      GLSLIRRWRSHD--VSLPVLVLTAREGWQDKVEVLSAGADDYVTKPFHIEEVAARMQALL   116
             *:::::: :.::    :**:****: * :*:  .** ** . : ***  .:.  .:: **

RR468        NE----------------------------------------------------------   122
PhoP_Kp      RRNSGL
```

Figure S1. ClustalW alignment between the REC domain of RR468 in PDB entry 6RFV and of PhoP in *K. pneumoniae* MGH 78578. Red, phosphorylatable aspartate (RR468, D53; PmrA, D51). Yellow, positions substituted in polymyxin resistant isolates carrying wt *mgrB*: G121A. Sequence identity between RR468 and PhoP REC domains is 32%.
