# Supplementary material for: Polymyxin Resistance in Clinical Isolates of K. pneumoniae in Brazil: Update on Molecular Mechanisms, Clonal Dissemination and Relationship With KPC-Producing Strains
Source: Front Cell Infect Microbiol. 2022 Jul 15;12:898125. doi: 10.3389/fcimb.2022.898125 (PMC9334684; doi:10.3389/fcimb.2022.898125)
Supplement: Supplementary file 2 [file Image_2.pdf]

```
RR468           MSKKVLLVDDSAVLRKIVSFNLKKEGYEVIEAENGQIALEKLSEFTPDLIVLDIMMPVMD    60
PmrA_Kp         --MKILVIEDDALLLQGLILAMQSEGYVCDGVSTAHEAALSLASNHYSLIVLDLGLPDED    58
                   *:*:::*.*:* : : : ::.***    ....: *  .*:.   .*****: :*  *

RR468           GFTVLKKLQEKEEWKRIPVIVLTAKGGEEDESLALSLGARKVMRKPFSPSQFIEEVKHLL    120
PmrA_Kp         GLHFLSRMRRE--KMTQPVLILTARDTLEDRISGLDTGADDYLVKPFALEELNARIRALL    116
                *: .*.:::.:      **::***:.  **.  .*. ** . : ***: .::  .:: **

RR468           NE----------------------------------------------------------    122
PmrA_Kp         RRHNNQ
```

Figure S2. ClustalW alignment between the REC domain of RR468 in PDB entry 6RFV and of PmrA in *K. pneumoniae* MGH 78578. Red, phosphorylatable aspartate (RR468, D53; PmrA, D51). Yellow, positions substituted in polymyxin resistant isolates carrying wt *mgrB*: A41T, L63H. Sequence identity between RR468 and PmrA REC domains is 29%.
