# Supplementary material for: Polymyxin Resistance in Clinical Isolates of K. pneumoniae in Brazil: Update on Molecular Mechanisms, Clonal Dissemination and Relationship With KPC-Producing Strains
Source: Front Cell Infect Microbiol. 2022 Jul 15;12:898125. doi: 10.3389/fcimb.2022.898125 (PMC9334684; doi:10.3389/fcimb.2022.898125)
Supplement: Supplementary file 3 [file Image_3.pdf]

```
HK853           ------------------------------------------------MEEIKEQNNRLF   12
PhoQ_Kp         MKGLLRHIFPLSLRVRFLLATAGVVLVLSLAYGMVALVGYSVSFDKTTFRLLRGESNLFY   60
PmrB_Kp         --------------------------------------------------------MALF   4
                                                          ::

HK853           LLLSIMIMEFSNAKTESDILQGLLNLVRKVVDIKEVVLVDENKRKIWGRDIDIKRFEEFI   72
PhoQ_Kp         M-----LARWENGAIDVDIPENL-NME----SPTVTLIYDEQGKLLWAQRD--------V   102
PmrB_Kp         A-----TETWTM-----------------------------RHRLLLTIGA--------I   22
                         :                               . : :             :

HK853           DWSIRQSNPVFVEDELGYVGIVPVVKQDRMF--------GSLIVL--LNHQPSMEETE--   120
PhoQ_Kp         PWLAKRIQPEWLKR-NGFHEIEADVDSSSMLLRNNHEIQEQLDAIREQGDDSEMTHSVAI   161
PmrB_Kp         LVVCQLISVFWLWH-ESKEQIQLLVAS----------------AIEGHNNQKHVEHEVRE   65
                    :  .  ::    .   *   * .                .:   ..:  : .

HK853           -IFKV---LSFLSAVVLENIKLYRELEETYNYVNVILNGLPEGIFVYSNGEIKFQNEKFK   176
PhoQ_Kp         NLYPATSKMPQLSIVVVDTIP--VELKRSYMVWSWFVYVLAANLLLVIPLL--WVAA--W   215
PmrB_Kp         A--VASLLVPSLLI---------------------------VGLALYISML----AV--R   90
                    .   :  *                              .: :

HK853           EENFPDEVLRKAISLSEEAISLRTQRVGEVISGEEFFSITSIPLILGSEVQALTIVENVT   236
PhoQ_Kp         WSLRPIESLAKEVR------EL-----------EEHHREKLNPNT----TRELTRLV---   251
PmrB_Kp         KITRPLSRLQ---S------EL-----------ENRTPDNLTPIVLSESVPEVTAVT---   127
                    * . *           .*           *:    .  *      .  :* :

HK853           ESKELERLK-----RIDRMKTEFIANISHELRTPLTAIKAYAETIYNSLGELDLSTLKEF   291
PhoQ_Kp         --SNLNRLVRSERERYD-KYRTTLTDLTHSLKTPLAVMQSTLRSLRGEK----IS-VDEA   303
PmrB_Kp         --TALNQLVSRLNLTLD-RERLFTADVAHELRTPLAGLRLHLELLAKVH-------GMGV   177
                  . *::*        *       ::::*.*:***: ::   . :

HK853           LEVIIDQSNHLENLLNELLDFSRLERKS--------LQINREKVDLCDLVESAVNAIKEF   343
PhoQ_Kp         EPVMLEQISRISQQIGYYLHRA----SMRSGG----TLLSRELHPIAPLLDSLTSALNKV   355
PmrB_Kp         DPL-IQRLDQMTTSISQLLQLARVGQSFSAGSYQQVLLLDDV---VKPLQDELEAMLA--   231
                  : ::: .::   :.  *. :    .           :.     :  * :.    :

HK853           ASSHNVNVLFESNVPCPVEAYIDPTRIRQVLLNLLNNGVKYSKKDAPDKYVKVILDEKDG   403
PhoQ_Kp         YQRKGVNISL--DISPEITFVGEQNDFMEVMGNVLDNACKYCLEFVEV----SVRQTTDS   409
PmrB_Kp         -QRQQRLLLT--DIENETVVSGDATLIRVILRNLVENAHRYSPEGSTI----RVSVKAGL   284
                 . :   :    ::        : . :  :: *:::*. :*. :         :    .

HK853           GVLIIVEDNGIGIPDHAKDRIFEQFYRVDSSLTYEVPGTGLGLAITKEIVELHGGRIWVE   463
PhoQ_Kp         HLHILVEDDGPGIPQSQRRAVFDRGQRADTL----RPGQGVGLSVAREIVEQYDGEIIAG   465
PmrB_Kp         MPVMAVEDEGPGIDEAKSGELSKAFVRMDSR----YGGIGLGLSIVTRIAQLHDAQFFLH   340
                   : ***:* ** :     : .   * *:       * *:**::. .*.: :...:

HK853           SE-VGKGSRFFVWIPKDRAGEDNRQDN       489
PhoQ_Kp         ESLLGGAC--ME-VVFGRQQMEDKQS-       488
PmrB_Kp         NRQPGPGV--RAWVLFPQRGGQNVSTH       365
                .   * .      :   :   :: .
```

Figure S3. ClustalW alignment between HK853, PhoQ and PmrB of *K. pneumoniae* MGH 78578. Bold: portion of HK853 in PDB structure 6RFN. Red, phosphorylatable histidine (HK853, H260; PhoQ, H277; PmrB, H153). Yellow, positions substituted in polymyxin resistant isolates carrying wt *mgrB*: PhoQ I88N, Y265C, Y265T, T276C, E397G, Q405A and PmrB H61Q, T240M, V280L, M285L. Sequence identity between proteins are as follows: PhoQ-PmrB, 26%; HK853-PhoQ, 22%; HK853-PmrB, 22%.
