# Supplementary material for: Polymyxin Resistance in Clinical Isolates of K. pneumoniae in Brazil: Update on Molecular Mechanisms, Clonal Dissemination and Relationship With KPC-Producing Strains
Source: Front Cell Infect Microbiol. 2022 Jul 15;12:898125. doi: 10.3389/fcimb.2022.898125 (PMC9334684; doi:10.3389/fcimb.2022.898125)
Supplement: Supplementary file 4 [file Table_1.pdf]

Supplementary table S1. Compiled data from 148 colistin-resistant *Klebsiella pneumoniae* clinical isolates from eight Brazilian states.

State	CCBH No.	Specimen	Collection date	Microorganism	Colistin MIC	*mcr*	*bla*$_{KPC}$	*bla*$_{NDM}$	*bla*$_{OXA48}$	PFGE	PmrA	PmrB	PhoP	PhoQ	MgrB	MLST
SE	21801	Rectal swab	11/01/2016	*Klebsiella pneumoniae*	>128	ND	Detectable	ND	ND	Kp41	E57G	WT	WT	WT	WT	*
MA	21849	Rectal swab	13/01/2016	*Klebsiella pneumoniae*	>128	ND	ND	ND	ND	Kp22	E57G	WT	WT	D73I	Not amplified	*
MA	21854	Tracheal aspirate	13/01/2016	*Klebsiella pneumoniae*	>128	ND	Detectable	ND	ND	Kp22	WT	T240M	WT	WT	Not amplified	*
MA	21875	Rectal swab	13/01/2016	*Klebsiella pneumoniae*	>128	ND	ND	ND	ND	Kp23	WT	T246A	WT	WT	WT	4868
ES	21941	Urine	08/01/2016	*Klebsiella pneumoniae*	>128	ND	Detectable	ND	ND	Kp22	WT	R256G, T246A	WT	WT	WT	STND
RS	22005	Tracheal aspirate	08/01/2016	*Klebsiella pneumoniae*	64	ND	Detectable	ND	ND	Kp51	WT	R256G, T246A	WT	WT	WT	*
RS	22053	Urine	09/01/2016	*Klebsiella pneumoniae*	>128	ND	ND	ND	ND	Kp14	E57G	T246A	WT	WT	WT	*
ES	22063	Tissue fragment	18/01/2016	*Klebsiella pneumoniae*	64	ND	Detectable	ND	ND	Kp6	WT	R256G, T246A	WT	WT	WT	*
RJ	22114	Blood	13/01/2016	*Klebsiella pneumoniae*	32	ND	Detectable	ND	ND	Kp38	S64A, N131D, L140Q, E199D	N105S, A228T, Q232E, I242V, N244S, T246A	WT	A69K, Q92K, A106T, E112D, I139V, L163F, V196I, Q424P	WT	526
RJ	22118	Urine	13/01/2016	*Klebsiella pneumoniae*	64	ND	Detectable	ND	ND	Kp6	WT	R256G, T246A	WT	WT	WT	*
RJ	22124	Urine	21/01/2016	*Klebsiella pneumoniae*	64	ND	Detectable	ND	ND	Kp27	WT	R256G, T246A	WT	WT	WT	76
RJ	22125	Rectal swab	21/01/2016	*Klebsiella pneumoniae*	>128	ND	Detectable	ND	ND	Kp27	WT	R256G, T246A	WT	WT	WT	*
RJ	22126	Rectal swab	21/01/2016	*Klebsiella pneumoniae*	32	ND	Detectable	ND	ND	Kp27	WT	R256G, T246A	WT	Ins1077A, Ins1078G	V1E, K2R, K3STOP	*
RJ	22128	Rectal swab	21/01/2016	*Klebsiella pneumoniae*	64	ND	Detectable	ND	ND	Kp27	WT	R256G, T246A	G121A	WT	WT	*

State	CCBH No.	Specimen	Collection date	Microorganism	Colistin MIC	*mcr*	*bla*KPC	*bla*NDM	*bla*OXA48	PFGE	PmrA	PmrB	PhoP	PhoQ	MgrB	MLST
RJ	22131	Rectal swab	21/01/2016	*Klebsiella pneumoniae*	32	ND	Detectable	ND	ND	Kp16	WT	WT	WT	WT	Not amplified	*
RJ	22133	Rectal swab	21/01/2016	*Klebsiella pneumoniae*	16	ND	Detectable	ND	ND	Kp40	WT	WT	WT	WT	WT	*
RJ	22134	Rectal swab	21/01/2016	*Klebsiella pneumoniae*	16	ND	Detectable	ND	ND	Kp27	WT	T246A, T240M	WT	WT	WT	*
RJ	22137	Tracheal aspirate	21/01/2016	*Klebsiella pneumoniae*	16	ND	Detectable	ND	ND	Kp40	WT	WT	WT	WT	ISKpn26	15
RJ	22138	Blood	21/01/2016	*Klebsiella pneumoniae*	>128	ND	Detectable	ND	ND	Kp16	WT	R256G, T246A	WT	WT	G37S	258
RJ	22139	Urine	21/01/2016	*Klebsiella pneumoniae*	64	ND	ND	ND	ND	Kp1	WT	T246A	WT	WT	WT	*
RJ	22143	Rectal swab	21/01/2016	*Klebsiella pneumoniae*	32	ND	Detectable	ND	ND	Kp2	WT	R256G, T246A	WT	I88N	WT	*
RJ	22146	Urine	27/01/2016	*Klebsiella pneumoniae*	32	ND	Detectable	ND	ND	Kp5	WT	R256G, T246A	WT	WT	WT	*
ES	22156	Urine	26/01/2016	*Klebsiella pneumoniae*	32	ND	Detectable	ND	ND	Kp50	WT	R256G, T246A	WT	WT	WT	*
SE	22168	Blood	03/02/2016	*Klebsiella pneumoniae*	64	ND	Detectable	ND	ND	Kp7	E57G	R256G, T246A	WT	WT	Not amplified	*
ES	22193	Urine	28/01/2016	*Klebsiella pneumoniae*	32	ND	Detectable	ND	ND	Kp50	WT	R256G, T246A	WT	WT	WT	*
SE	22206	Rectal swab	25/01/2016	*Klebsiella pneumoniae*	64	ND	Detectable	ND	ND	Kp7	WT	R256G, T246A	WT	V27H, P103W, C395A	ISKpn13	*
SE	22208	Rectal swab	11/02/2016	*Klebsiella pneumoniae*	64	ND	Detectable	ND	ND	Kp7	WT	R256G, T246A	WT	WT	WT	*
ES	22220	Tracheal aspirate	01/02/2016	*Klebsiella pneumoniae*	8	mcr-1 +	ND	ND	ND	Kp21	WT	R256G, T246A	WT	WT	WT	*
RJ	22225	Rectal swab	02/02/2016	*Klebsiella pneumoniae*	4	ND	Detectable	ND	ND	Kp31	WT	R256G, T246A	WT	Ins1077A, Ins1078G	WT	987
RJ	22237	Urine	29/01/2016	*Klebsiella pneumoniae*	32	ND	Detectable	ND	ND	Kp6	WT	R256G, T246A	WT	WT	IS1R	*
RJ	22240	Urine	29/01/2016	*Klebsiella pneumoniae*	32	ND	Detectable	ND	ND	Kp5	WT	R256G, T246A	WT	WT	IS903B	*

State	CCBH No.	Specimen	Collection date	Microorganism	Colistin MIC	*mcr*	*bla*KPC	*bla*NDM	*bla*OXA48	PFGE	PmrA	PmrB	PhoP	PhoQ	MgrB	MLST
RS	22303	Rectal swab	02/01/2016	*Klebsiella pneumoniae*	>128	ND	Detectable	ND	ND	Kp43	WT	R256G, T246A	WT	WT	WT	437
RS	22306	Rectal swab	05/01/2016	*Klebsiella pneumoniae*	>128	ND	Detectable	ND	ND	Kp3	WT	R256G, T246A	WT	WT	WT	*
ES	22367	Urine	25/02/2016	*Klebsiella pneumoniae*	4	ND	ND	ND	ND	Kp50	WT	R256G, T246A	WT	WT	WT	*
RJ	22381	Rectal swab	19/02/2016	*Klebsiella pneumoniae*	64	ND	ND	ND	ND	Kp36	WT	T246A	WT	WT	WT	Inviável
RJ	22382	Rectal swab	19/02/2016	*Klebsiella pneumoniae*	64	ND	Detectable	ND	ND	Kp20	WT	T246A	WT	WT	Ins 37pb between position 62 and 63	Inviável
RJ	22389	Rectal swab	04/02/2016	*Klebsiella pneumoniae*	>128	ND	Detectable	ND	ND	Kp1	WT	R256G, T246A	WT	WT	WT	*
RJ	22391	Urine	04/02/2016	*Klebsiella pneumoniae*	32	ND	Detectable	ND	ND	Kp5	WT	R256G, T246A	WT	WT	IS903B	*
RJ	22397	Urine	16/02/2016	*Klebsiella pneumoniae*	64	ND	ND	ND	ND	Kp14	E57G	T246A	WT	V27H, Y265C	WT	*
RJ	22399	Blood	15/02/2016	*Klebsiella pneumoniae*	64	mcr-1 +	ND	ND	ND	Kp21	WT	WT	WT	T276C	WT	15
SE	22404	Rectal swab	29/01/2016	*Klebsiella pneumoniae*	4	ND	ND	Detectable	ND	Kp41	E57G	R256G, H61Q, T246A	WT	WT	WT	STND
SE	22407	Rectal swab	15/01/2016	*Klebsiella pneumoniae*	8	ND	ND	Detectable	ND	Kp30	WT	R256G, T246A	WT	WT	WT	*
SE	22408	Rectal swab	30/01/2016	*Klebsiella pneumoniae*	16	ND	ND	ND	ND	Kp45	WT	R256G, T246A	WT	Q405A	WT	*
RS	22459	Urine	17/01/2016	*Klebsiella pneumoniae*	8	ND	ND	ND	ND	Kp39	WT	WT	WT	WT	WT	*
RS	22462	Urine	18/01/2016	*Klebsiella pneumoniae*	16	ND	Detectable	ND	ND	Kp11	WT	R256G, T246A	WT	WT	S36R	*
RS	22466	Wound	13/01/2016	*Klebsiella pneumoniae*	64	ND	Detectable	ND	ND	Kp11	WT	R256G, T246A	WT	WT	S36R	*
RS	22470	Urine	03/03/2016	*Klebsiella pneumoniae*	32	ND	Detectable	ND	ND	Kp11	WT	WT	WT	Ins_765C	WT	*

State	CCBH No.	Specimen	Collection date	Microorganism	Colistin MIC	*mcr*	*bla*KPC	*bla*NDM	*bla*OXA48	PFGE	PmrA	PmrB	PhoP	PhoQ	MgrB	MLST
RS	22481	Tracheal aspirate	23/01/2016	*Klebsiella pneumoniae*	64	ND	Detectable	ND	ND	Kp11	WT	R256G, T246A	WT	WT	IS903B	*
RS	22491	Rectal swab	17/01/2016	*Klebsiella pneumoniae*	32	ND	Detectable	ND	ND	Kp3	WT	R256G, T246A	WT	WT	ISKpn13	*
RJ	22609	Urine	26/02/2016	*Klebsiella pneumoniae*	64	mcr-1 +	Detectable	ND	ND	Kp21	WT	T246A	WT	V27H, Y265T	WT	*
RJ	22614	Tracheal aspirate	26/02/2016	*Klebsiella pneumoniae*	128	ND	Detectable	ND	ND	Kp4	WT	R256G, T246A	WT	V27H	WT	*
RJ	22615	Urine	25/02/2016	*Klebsiella pneumoniae*	128	ND	Detectable	ND	ND	Kp6	WT	R256G, T246A	WT	V27H	WT	*
RJ	22625	Tracheal aspirate	01/03/2016	*Klebsiella pneumoniae*	64	ND	Detectable	ND	ND	Kp5	WT	R256G, T246A	WT	WT	ISKpn26	*
ES	22632	Urine	05/03/2016	*Klebsiella pneumoniae*	64	ND	Detectable	ND	ND	Kp50	WT	R256G, T246A	WT	WT	WT	437
SE	22648	Rectal swab	25/01/2016	*Klebsiella pneumoniae*	64	ND	ND	ND	ND	Kp8	WT	R256G, T246A	WT	WT	WT	340
SE	22652	Rectal swab	29/02/2016	*Klebsiella pneumoniae*	32	ND	Detectable	ND	ND	Kp49	WT	R256G, T246A	WT	D73I	WT	*
SE	22653	Rectal swab	14/03/2016	*Klebsiella pneumoniae*	128	ND	Detectable	ND	ND	Kp7	WT	L222A	WT	WT	ISKpn13	11
RJ	22675	Rectal swab	15/03/2016	*Klebsiella pneumoniae*	64	ND	Detectable	ND	ND	Kp14	WT	T246A	WT	Y265C	WT	*
RJ	22678	Urine	15/03/2016	*Klebsiella pneumoniae*	64	ND	Detectable	ND	ND	Kp24	WT	R256G, T246A	WT	WT	WT	437
RJ	22679	Rectal swab	15/03/2016	*Klebsiella pneumoniae*	64	ND	ND	ND	Detectable	Kp14	E57G	T246A	WT	WT	Not amplified	*
RJ	22680	Rectal swab	15/03/2016	*Klebsiella pneumoniae*	64	ND	Detectable	ND	ND	Kp35	WT	T246A	WT	WT	WT	4869
RJ	22684	Rectal swab	09/03/2016	*Klebsiella pneumoniae*	32	ND	Detectable	ND	ND	Kp9	WT	R256G, T246A	WT	WT	WT	437
RS	22723	Blood	04/01/2016	*Klebsiella pneumoniae*	>128	ND	Detectable	ND	ND	Kp37	WT	WT	WT	WT	WT	STND
RS	22733	Tracheal aspirate	21/02/2016	*Klebsiella pneumoniae*	32	ND	Detectable	ND	ND	Kp3	WT	WT	WT	WT	WT	*
RS	22736	Blood	17/02/2016	*Klebsiella pneumoniae*	>128	ND	Detectable	ND	ND	Kp3	WT	WT	WT	WT	WT	*

State	CCBH No.	Specimen	Collection date	Microorganism	Colistin MIC	*mcr*	$\mathit{bla}_{\mathrm{KPC}}$	$\mathit{bla}_{\mathrm{NDM}}$	$\mathit{bla}_{\mathrm{OXA48}}$	PFGE	PmrA	PmrB	PhoP	PhoQ	MgrB	MLST
RS	22737	Blood	17/02/2016	*Klebsiella pneumoniae*	>128	ND	Detectable	ND	ND	Kp3	WT	R256G, T246A	WT	WT	WT	*
RS	22740	Tracheal aspirate	02/02/2016	*Klebsiella pneumoniae*	64	ND	Detectable	ND	ND	Kp51	WT	R256G, T246A	WT	E397G	WT	*
RS	22760	Blood	17/03/2016	*Klebsiella pneumoniae*	4	ND	Detectable	ND	ND	Kp51	WT	R256G, T246A	WT	WT	WT	11
MA	22871	Tracheal aspirate	30/03/2016	*Klebsiella pneumoniae*	8	ND	Detectable	ND	ND	Kp47	WT	E272Q, T246A, N244S, I242V, Q232E, A228T, N105S	WT	L163F, V196I, A325V	WT	477
MA	22890	Rectal swab	30/03/2016	*Klebsiella pneumoniae*	>128		Detectable	ND	ND	Kp45	WT	WT	WT	WT	WT	15
MA	22891	Blood	30/03/2016	*Klebsiella pneumoniae*	>128	ND	Detectable	ND	ND	Kp46	WT	WT	WT	WT	WT	15
MA	22912	Blood	30/03/2016	*Klebsiella pneumoniae*	16	ND	Detectable	ND	ND	Kp15	WT	R256G, T246A	WT	V27H, P103W	Not amplified	*
SE	22997	Rectal swab	23/03/2016	*Klebsiella pneumoniae*	16	ND	Detectable	ND	ND	Kp49	WT	R256G, T246A	WT	V27H, F398K	IS102	*
SE	22998	Rectal swab	23/03/2016	*Klebsiella pneumoniae*	>128	ND	ND	Detectable	ND	Kp49	WT	Ins_748C	WT	WT	Not amplified	*
SE	22999	Rectal swab	28/03/2016	*Klebsiella pneumoniae*	32	ND	Detectable	ND	ND	Kp49	WT	A282R, R256G, T246A	WT	WT	IS102	147
SE	23000	Rectal swab	28/03/2016	*Klebsiella pneumoniae*	16	ND	Detectable	ND	ND	Kp49	WT	A282R, R256G, T246A	WT	R16C	IS102	*
SE	23001	Rectal swab	25/03/2016	*Klebsiella pneumoniae*	32	ND	Detectable	ND	ND	Kp49	WT	R256G, T246A	WT	WT	IS102	*
RS	23014	Rectal swab	17/01/2016	*Klebsiella pneumoniae*	8	ND	Detectable	ND	ND	Kp11	WT	R256G, T246A	WT	WT	WT	*
RS	23015	Rectal swab	11/01/2016	*Klebsiella pneumoniae*	8	ND	Detectable	ND	ND	Kp11	WT	R256G, T246A	WT	WT	WT	*

State	CCBH No.	Specimen	Collection date	Microorganism	Colistin MIC	*mcr*	*bla*KPC	*bla*NDM	*bla*OXA48	PFGE	PmrA	PmrB	PhoP	PhoQ	MgrB	MLST
RS	23016	Rectal swab	22/01/2016	*Klebsiella pneumoniae*	4	ND	Detectable	ND	ND	Kp19	WT	R256G, T246A	WT	WT	WT	*
RS	23017	Urine	18/01/2016	*Klebsiella pneumoniae*	4	ND	Detectable	ND	ND	Kp51	WT	A282R, R256G, T246A	WT	WT	WT	*
RS	23024	Urine	28/01/2016	*Klebsiella pneumoniae*	32	ND	Detectable	ND	ND	Kp11	WT	R256G, T246A	WT	V27H, P103W, C395A	ISKpn26	*
RS	23026	Sputum	01/02/2016	*Klebsiella pneumoniae*	64	ND	Detectable	ND	ND	Kp3	WT	R256G, T246A	WT	WT	WT	*
RS	23030	Rectal swab	25/01/2016	*Klebsiella pneumoniae*	128	ND	Detectable	ND	ND	Kp3	WT	R256G, T246A	100_101ins 11pb	V27H, P103W	WT	*
RS	23031	Rectal swab	28/01/2016	*Klebsiella pneumoniae*	64	ND	Detectable	ND	ND	Kp11	WT	A282R, R256G, T246A	WT	V27H, P103W, C395A	IS102	*
RS	23033	Blood	28/01/2016	*Klebsiella pneumoniae*	64	ND	Detectable	ND	ND	Kp11	WT	A282R, R256G, T246A	WT	WT	WT	437
RS	23043	Rectal swab	27/01/2016	*Klebsiella pneumoniae*	32	ND	Detectable	ND	ND	Kp11	WT	R256G, T246A	WT	V27H, P103W, C395A	S36R	*
RS	23048	Urine	27/01/2016	*Klebsiella pneumoniae*	32	ND	Detectable	ND	ND	Kp11	WT	A282R, R256G, T246A	WT	WT	S36R	*
RS	23050	Rectal swab	26/01/2016	*Klebsiella pneumoniae*	32	ND	Detectable	ND	ND	Kp11	WT	R256G, T246A	WT	P103W, C395A	S36R	*
RS	23053	Rectal swab	18/01/2016	*Klebsiella pneumoniae*	32	ND	Detectable	ND	ND	Kp14	WT	T246A	WT	V27H, D73I, P103W, S188T, C395A	WT	*
RS	23058	Rectal swab	22/01/2016	*Klebsiella pneumoniae*	16	ND	Detectable	ND	ND	Kp51	WT	R256G, T246A	WT	V27H	WT	*
RS	23064	Rectal swab	21/01/2016	*Klebsiella pneumoniae*	128	ND	Detectable	ND	ND	Kp11	WT	R256G, T246A	WT	WT	IS903B	*

State	CCBH No.	Specimen	Collection date	Microorganism	Colistin MIC	*mcr*	*bla*$_{KPC}$	*bla*$_{NDM}$	*bla*$_{OXA48}$	PFGE	PmrA	PmrB	PhoP	PhoQ	MgrB	MLST
RS	23065	Urine	24/01/2016	*Klebsiella pneumoniae*	64	ND	Detectable	ND	ND	Kp11	WT	R256G, T246A	WT	WT	WT	*
RS	23068	Tracheal aspirate	20/01/2016	*Klebsiella pneumoniae*	16	ND	Detectable	ND	ND	Kp11	WT	R256G, T246A	WT	WT	WT	*
RS	23069	Urine	25/01/2016	*Klebsiella pneumoniae*	16	ND	Detectable	ND	ND	Kp11	WT	R256G, T246A	WT	V27H, P103W	Not amplified	*
RS	23071	Rectal swab	25/01/2016	*Klebsiella pneumoniae*	16	ND	Detectable	ND	ND	Kp11	WT	R256G, T246A	WT	WT	WT	*
RS	23093	Tracheal aspirate	31/01/2016	*Klebsiella pneumoniae*	4	ND	Detectable	ND	ND	Kp11	WT	R256G, T246A	WT	WT	WT	*
RS	23094	Urine	06/02/2016	*Klebsiella pneumoniae*	32	ND	Detectable	ND	ND	Kp12	WT	R256G, T246A	WT	P103W	WT	437
RS	23096	Rectal swab	27/01/2016	*Klebsiella pneumoniae*	32	ND	Detectable	ND	ND	Kp11	WT	R256G, T246A	WT	WT	WT	*
RS	23097	Tracheal aspirate	27/01/2016	*Klebsiella pneumoniae*	64	ND	Detectable	ND	ND	Kp14	R160S, E57G	T246A	WT	R16A, V27H, D73I, P103W, S188T	IS903B	*
RS	23110	Blood	27/01/2016	*Klebsiella pneumoniae*	64	ND	Detectable	ND	ND	Kp3	WT	R256G, T246A	WT	WT	WT	11
RS	23123	Urine	04/02/2016	*Klebsiella pneumoniae*	16	ND	Detectable	ND	ND	Kp3	WT	R256G, T246A	WT	WT	WT	*
RS	23129	Sputum	15/02/2016	*Klebsiella pneumoniae*	4	ND	Detectable	ND	ND	Kp44	E57G	R256G, T246A	WT	WT	WT	16
RS	23132	Rectal swab	08/02/2016	*Klebsiella pneumoniae*	32	ND	Detectable	ND	ND	Kp12	WT	R256G, T246A	WT	WT	WT	*
RS	23134	Rectal swab	08/02/2016	*Klebsiella pneumoniae*	8	ND	Detectable	ND	ND	Kp12	WT	R256G, T246A	WT	WT	WT	*
RS	23138	Rectal swab	08/02/2016	*Klebsiella pneumoniae*	4	ND	Detectable	ND	ND	Kp2	WT	R256G, T246A	WT	WT	Not amplified	*
RS	23145	Blood	15/02/2016	*Klebsiella pneumoniae*	32	ND	Detectable	ND	ND	Kp10	WT	R256G, T246A	WT	V27H, P103W, C395A	WT	437

State	CCBH No.	Specimen	Collection date	Microorganism	Colistin MIC	*mcr*	*bla*KPC	*bla*NDM	*bla*OXA48	PFGE	PmrA	PmrB	PhoP	PhoQ	MgrB	MLST
RS	23161	Urine	10/02/2016	*Klebsiella pneumoniae*	16	ND	Detectable	ND	ND	Kp3	WT	R256G, T246A	WT	WT	WT	*
RS	23164	Rectal swab	27/01/2016	*Klebsiella pneumoniae*	8	ND	Detectable	ND	ND	Kp11	WT	R256G, T246A	WT	WT	WT	*
PI	23167	Rectal swab	18/03/2016	*Klebsiella pneumoniae*	16	ND	Detectable	ND	ND	Kp26	A41T	T246A, L213M	WT	C395V	WT	STND
ES	23171	Urine	25/03/2016	*Klebsiella pneumoniae*	32	ND	Detectable	ND	ND	Kp13	WT	T246A	WT	WT	K3STOP	111
MA	23220	Blood	06/04/2016	*Klebsiella pneumoniae*	>128	ND	Detectable	ND	ND	Kp15	WT	T246A, M285L	WT	WT	WT	3228
MA	23247	Rectal swab	06/04/2016	*Klebsiella pneumoniae*	32	ND	Detectable	ND	ND	Kp24	WT	L213M, T246A	WT	WT	IS903B	*
MA	23279	Tissue fragment	05/04/2016	*Klebsiella pneumoniae*	4	ND	Detectable	ND	ND	Kp17	WT	R256G, T246A	WT	WT	WT	617
MA	23286	Tracheal aspirate	05/04/2016	*Klebsiella pneumoniae*	4	ND	Detectable	ND	ND	Kp30	E57G	N105S, A228T, Q232E, I242V, N244S, T246A, E272Q	WT	R64K, Q92K, A106T, E112D, I139V, L163F, V196I, T372S, Q424P	WT	477
RS	23296	Sputum	03/02/2016	*Klebsiella pneumoniae*	64	ND	Detectable	ND	ND	Kp51	WT	R256G, T246A	WT	WT	ISKpn13	*
RS	23300	Wound	17/02/2016	*Klebsiella pneumoniae*	128	ND	Detectable	ND	ND	Kp11	WT	R256G, T246A	WT	WT	Not amplified	*
RS	23304	Tracheal aspirate	15/02/2016	*Klebsiella pneumoniae*	4	ND	Detectable	ND	ND	Kp14	E57G	T246A	WT	WT	Not amplified	*
RS	23308	Blood	19/02/2016	*Klebsiella pneumoniae*	32	ND	Detectable	ND	ND	Kp28	E57G	R256G, T246A	WT	WT	WT	16
RS	23323	Tracheal aspirate	23/02/2016	*Klebsiella pneumoniae*	>128	ND	Detectable	ND	ND	Kp3	L63H	R256G	WT	WT	WT	*

State	CCBH No.	Specimen	Collection date	Microorganism	Colistin MIC	*mcr*	*bla*$_{KPC}$	*bla*$_{NDM}$	*bla*$_{OXA48}$	PFGE	PmrA	PmrB	PhoP	PhoQ	MgrB	MLST
RS	23333	Tissue fragment	24/02/2016	*Klebsiella pneumoniae*	>128	ND	Detectable	ND	ND	Kp11	WT	R256G, T246A	WT	L257W, Q287K, Ins878A, Ins879G	WT	*
RS	23336	Urine	25/02/2016	*Klebsiella pneumoniae*	64	ND	Detectable	ND	ND	Kp11	WT	R256G, T246A	WT	WT	Not amplified	*
RS	23337	Rectal swab	28/02/2016	*Klebsiella pneumoniae*	4	ND	Detectable	ND	ND	Kp3	WT	R256G, T246A	WT	WT	WT	*
RS	23347	Rectal swab	14/02/2016	*Klebsiella pneumoniae*	8	ND	ND	ND	ND	Kp25	WT	T246A	WT	V27H, P103W, C395A	WT	252
RS	23357	Blood	27/02/2016	*Klebsiella pneumoniae*	16	ND	ND	ND	ND	Kp19	WT	R256G, T246A	WT	WT	WT	437
PI	23368	Catheter tip	26/03/2016	*Klebsiella pneumoniae*	4	ND	Detectable	ND	ND	Kp4	WT	R256G, T246A	WT	WT	K3T	*
GO	23421	Blood	31/01/2016	*Klebsiella pneumoniae*	32	ND	ND	ND	ND	Kp5	WT	V280L, T246A	WT	WT	WT	48
GO	23432	Tracheal aspirate	30/03/2016	*Klebsiella pneumoniae*	8	ND	ND	ND	ND	Kp33	WT	R256G, T246A	WT	WT	WT	340
GO	23454	Tracheal aspirate	30/03/2016	*Klebsiella pneumoniae*	16	ND	Detectable	ND	ND	Kp34	WT	V280L, T246A	WT	WT	WT	48
GO	23462	Catheter tip	04/05/2016	*Klebsiella pneumoniae*	>128	ND	Detectable	ND	ND	Kp4	WT	R256G, T246A, S203G	WT	WT	I10T	11
MG	23504	Urine	09/03/2016	*Klebsiella pneumoniae*	>128	ND	ND	ND	ND	Kp39	WT	T246A	WT	WT	WT	101
MG	23510	Blood	22/02/2016	*Klebsiella pneumoniae*	32	ND	Detectable	ND	ND	Kp29	WT	R256G, T246A, Ins_534T	WT	V27H, Ins_844C, F398K	IS903B	147
MG	23512	Tracheal aspirate	21/02/2016	*Klebsiella pneumoniae*	128	ND	ND	ND	ND	Kp4	WT	R256G, T246A	WT	WT	WT	*
GO	23613	Urine	16/06/2016	*Klebsiella pneumoniae*	64	ND	Detectable	ND	ND	Kp1	WT	R256G, T246A	WT	WT	Not amplified	*

State	CCBH No.	Specimen	Collection date	Microorganism	Colistin MIC	*mcr*	*bla*KPC	*bla*NDM	*bla*OXA48	PFGE	PmrA	PmrB	PhoP	PhoQ	MgrB	MLST
GO	23614	Tracheal aspirate	22/06/2016	*Klebsiella pneumoniae*	128	ND	Detectable	ND	ND	Kp2	WT	R256G, T246A	WT	WT	Not amplified	*
GO	23615	Urine	22/06/2016	*Klebsiella pneumoniae*	>128	ND	Detectable	ND	ND	Kp2	WT	R256G, T246A	WT	WT	ISKpn26 Nt65	*
GO	23616	Blood	22/06/2016	*Klebsiella pneumoniae*	128	ND	Detectable	ND	ND	Kp2	WT	R256G, T246A	WT	WT	WT	258
GO	23617	Rectal swab	24/06/2016	*Klebsiella pneumoniae*	4	ND	ND	ND	ND	Kp19	WT	R256G, T246A	WT	WT	WT	*
MG	23650	Tissue fragment	15/03/2016	*Klebsiella pneumoniae*	64	ND	Detectable	ND	ND	Kp42	E57G	G345R, T246A	WT	WT	L8STOP	17
MG	23651	Urine	01/02/2016	*Klebsiella pneumoniae*	64	ND	ND	ND	ND	Kp48	WT	WT	WT	WT	WT	15
MG	23661	Rectal swab	08/04/2016	*Klebsiella pneumoniae*	>128	ND	ND	ND	ND	Kp29	WT	R256G, T246A	WT	WT	IS903B	*
MG	23663	Urine	27/04/2016	*Klebsiella pneumoniae*	>128	ND	Detectable	ND	ND	Kp29	WT	R256G, T246A	WT	WT	IS903B	*
ES	23707	Urine	24/04/2016	*Klebsiella pneumoniae*	>128	ND	Detectable	ND	ND	Kp32	WT	WT	WT	WT	WT	54
ES	23711	Urine	28/04/2016	*Klebsiella pneumoniae*	>128	ND	Detectable	ND	ND	Kp6	WT	R256G, T246A	WT	WT	WT	*
ES	23736	Urine	06/06/2016	*Klebsiella pneumoniae*	16	ND	Detectable	ND	ND	Kp18	WT	WT	WT	WT	Not amplified	11
ES	23741	Blood	16/06/2016	*Klebsiella pneumoniae*	128	ND	Detectable	ND	ND	Kp1	WT	R256G, T246A	WT	WT	IS903B	340
ES	23791	Blood	15/07/2016	*Klebsiella pneumoniae*	>128	ND	ND	ND	Detectable	Kp14	E57G	T246A	WT	WT	WT	16
ES	23834	Urine	16/08/2016	*Klebsiella pneumoniae*	64	ND	Detectable	ND	ND	Kp6	WT	R256G, T246A	WT	WT	WT	437

Notes: In green font, neutral mutations according to the Provean algorithm. Deleterious mutations are in red font. WT= wild type; ND= not detected; STND= Sequence type not detected; * = not selected for MLST analysis.
